# Atrial fibrosis segmentation thresholds: a theoretical and empirical study

**DOI:** 10.1186/1532-429X-18-S1-P209

**Published:** 2016-01-27

**Authors:** Dana C Peters, Litten Bertelsen, Ita Caroline, Sudhakar Chelikani

**Affiliations:** 1 grid.47100.320000000419368710Diagnostic Radiology, Yale School of Medicine, New Haven, CT USA; 2 grid.5254.6000000010674042XCardiology, 2Department of Cardiology, The Heart Centre, Rigshospitalet, University of Copenhagenpen, Copenhagen, Denmark

## Background

High resolution late gadolinium enhancement (LGE) images of the left atrium (LA) are currently being used as evidence of atrial fibrosis [1], and their correlation to electroanatomic voltage mapping has been analyzed at multiple signal thresholds [2,3], e.g. using CNR or enhancement ratio (ER, LA wall to mean blood signal). However, the true correlate to the clinical and physiological metrics of atrial remodeling, such as voltage, is the collagen extent, or the extra-cellular volume fraction (ECV). Therefore, the optimal method (CNR or ER) and the optimal threshold values for segmenting scar is unclear, despite the several studies. CNR=(S_wall_-S_b_)/_b_ and ER=S_wall_/S_b_, and therefore they are mathematically related via blood SNR, as ER=CNR/SNR_b_+1. Although related, use of ER vs. CNR represent different approaches to segmentation. On one hand, ER is the ratio of scar to blood signal, and therefore related to the ratio of contrast agent concentration in wall and blood—ergo ECV. On the other, choosing a threshold based on CNR allows a trade-off between sensitivity and specificity for selecting enhanced pixels. Here we studied the optimal ER and CNR for predicting lower bipolar voltage.

## Methods

ER was simulated using Bloch Equations for various post-contrast [T1b, T1_wall_ ] combinations, assuming scan parameters of TR/θ/vps/RR=5.2 ms/20°/32/1000, TI set to null normal myocardium, assuming centric acquisition, no proton density or coil effects, 1RR per inversion. T1 combinations were converted to ECV values using average values T1^b^_0_=1500 ms, and T1^m^_0_=900 ms, and HCT=0.45. Eight atrial fibrillation patients with bipolar voltage mapping and pre-ablation LGE were studied. The voltage data was registered in 3D to the LGE signal intensities of the atrial myocardium [4]. The voltage data was fused with the LGE data, by landmark registration of the PV ostia identified in each data set. The voltage data was projected onto the MR derived LA wall. For each subject, 3D visualizations of voltage and LGE signal, and a plot of voltage vs. CNR and ER were generated.

## Results

**Figure 1:**
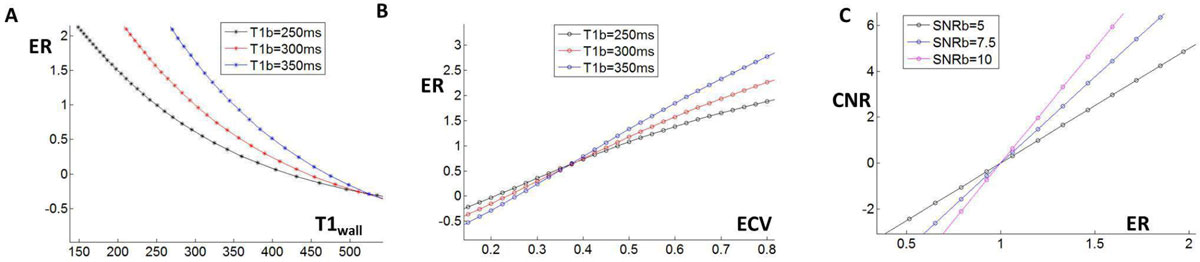
**Full Bloch simulations of LGE estimate blood and wall signal for input T1 values**. A) Calculated enhancement ratio (ER) vs. T1_wall_ for 3 blood T1 values. B) ERS vs. extra-cellular volume fraction (ECV), showing an almost linear relationship, which, depends on T1b. An ER or 1.4 correlates with an ACV of 55% for a 300 ms T1b. C) CNR vs. ER for various SNRb values.

**Figure 2:**
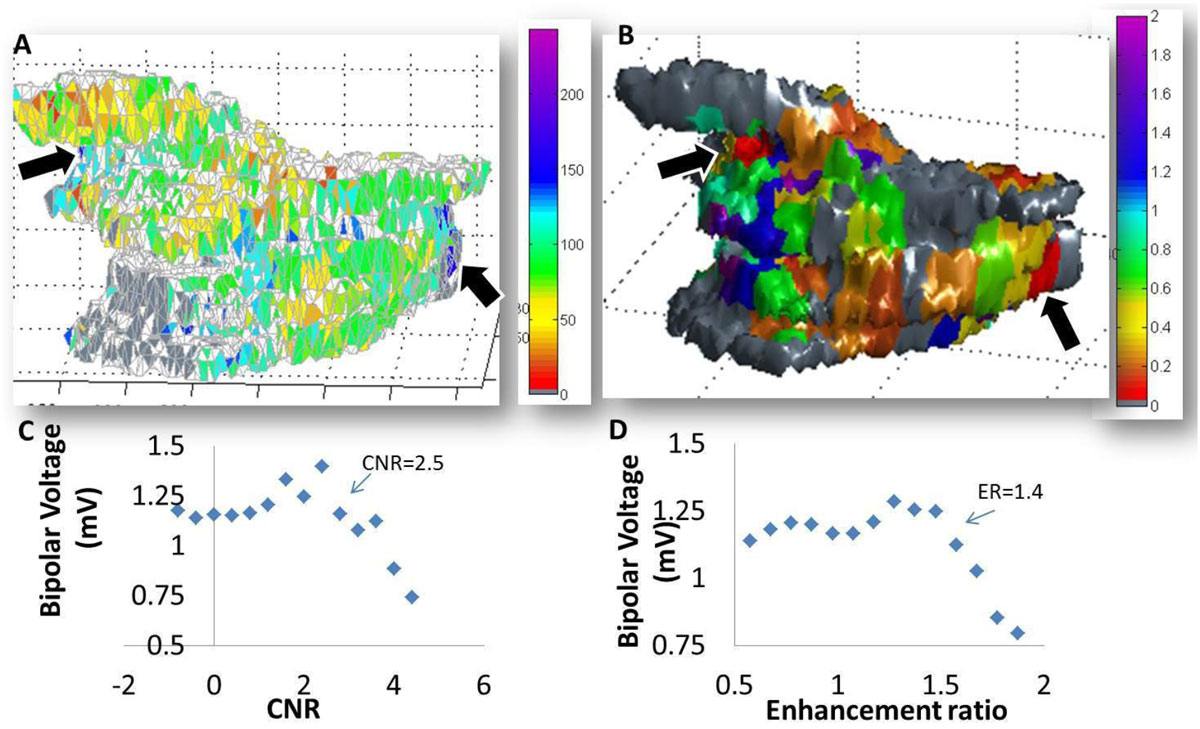
**A) 3D maps of left atrial enhancement ratio (ER in %) compared to B) bipolar voltage (in mV) in one patient**. Arrows indicate regions of highest ER (blue) matched with lowest voltage (red). C-D) After fusion of bipolar voltage maps with LGE signal intensity maps, the voltage at each pixel was compared to the corresponding CNR and ER (averaged over 8 subjects). C) CNR vs. voltage. B) ER vs. voltage). The trend displays a “knee” shape, after which high intensity corresponding to lower voltage.

Figure 1 show how ER depends on T1_wall_, T1b combinations, and how CNR scales with ER. Note that ER is somewhat linear with ECV. An ER threshold of 1.4 identifies ECV>55% for a typical blood value of 300 ms. Figure 2A-B compares bipolar voltage map and the LGE ER map, with good agreement in low voltage regions (arrows). Figure 2C-D shows plots of CNR and ER vs. voltage averaged over all 8 patients, showing an evident "knee" beyond which higher ER and CNR was correlated with lower voltage. In our patients the mean blood pool SNR was 6.1 ± 1.1. The optimal ER cutoff was 1.4 ± 0.2 (range 1.7 to 1.1), and the optimal CNR cutoff was 2.5 ± 0.7 (range 1.2 to 3.6).

## Conclusions

Both ER and CNR should be considered in choice of a threshold. ER has a linear relationship with ECV. Our findings suggest that use of ER >1.4 is a reasonable threshold for atrial fibrosis segmentation. However, at low blood SNR (<5), an ER of 1.4 results in a CNR <2, which will result in segmentation errors.
